# New Insight into Pseudo-Thermal Convection in Vibrofluidised Granular Systems

**DOI:** 10.1038/s41598-018-31152-8

**Published:** 2018-08-27

**Authors:** C. R. K. Windows-Yule, E. Lanchester, D. Madkins, D. J. Parker

**Affiliations:** 1 School of Chemical Engineering, The University of Birmingham, Edgbaston, Birmingham, B15 2TT UK; 2 Institute for Multi-Scale Simulation, Friedrich-Alexander Universität Erlangen-Nürnberg, Schloßplatz 4, 91054 Erlangen, Germany; 3 School of Physics and Astronomy, The University of Birmingham, Edgbaston, Birmingham, B15 2TT UK

## Abstract

Utilising a combination of experimental results obtained via positron emission particle tracking (PEPT) and numerical simulations, we study the influence of a system’s geometric and elastic properties on the convective behaviours of a dilute, vibrofluidised granular assembly. Through the use of a novel, ‘modular’ system geometry, we demonstrate the existence of several previously undocumented convection-inducing mechanisms and compare their relative strengths across a broad, multi-dimensional parameter space, providing criteria through which the dominant mechanism within a given system – and hence its expected dynamics – may be predicted. We demonstrate a range of manners through which the manipulation of a system’s geometry, material properties and imposed motion may be exploited in order to induce, suppress, strengthen, weaken or even invert granular convection. The sum of our results demonstrates that boundary-layer effects due to wall (in)elasticity or directional impulses due to ‘rough’ boundaries exert only a secondary influence on the system’s behaviour. Rather, the direction and strength of convective motion is predominantly determined by the energy flux in the vicinity of the system’s lateral boundaries, demonstrating unequivocally that pseudo-thermal granular convection is decidedly a collective phenomenon.

## Introduction

## Granular Convection

Convection within dry granular media is a subject of considerable scientific interest^1–5^, playing crucial rôles in fascinating phenomena such as segregation^6,7^, mixing^8^, pattern formation^9^ and granular capillarity^10^. Outside of academia, it is also vital in various industrial apparatus such as agitators^11^ heaters and dryers^12^ and mixers^13^, all of which are widely used across a diverse range of industries.

Granular convection is driven by differing mechanisms dependent on the density of the system in question^14^. Specifically, it is generally accepted that convective processes in systems with higher packing densities are driven predominantly by frictional effects^15^, while convection in more dilute systems is often referred to as ‘*pseudo-thermal*’ of ‘*buoyancy-driven*’^16–18^. The frictionally-driven convection exhibited by the former systems has been extensively studied for more than a century^19^, proffering a comparatively strong understanding of the phenomenon, and thus arming researchers and industrialists with a clear knowledge of the manners in which such convective motion may be controlled. For such densely-packed systems, a variety of manners of controlling said frictionally-driven convection have been successfully demonstrated^1,15,20–23^.

Conversely, there exists a comparatively small volume of research concerning more dilute systems which, consequently, remain incompletely understood^5^ and thus difficult to predict and control–a potentially significant issue both in academia and industry. As such, it is upon these systems that we focus presently.

## Convection in Dilute Granular Media

**Figure 1:**
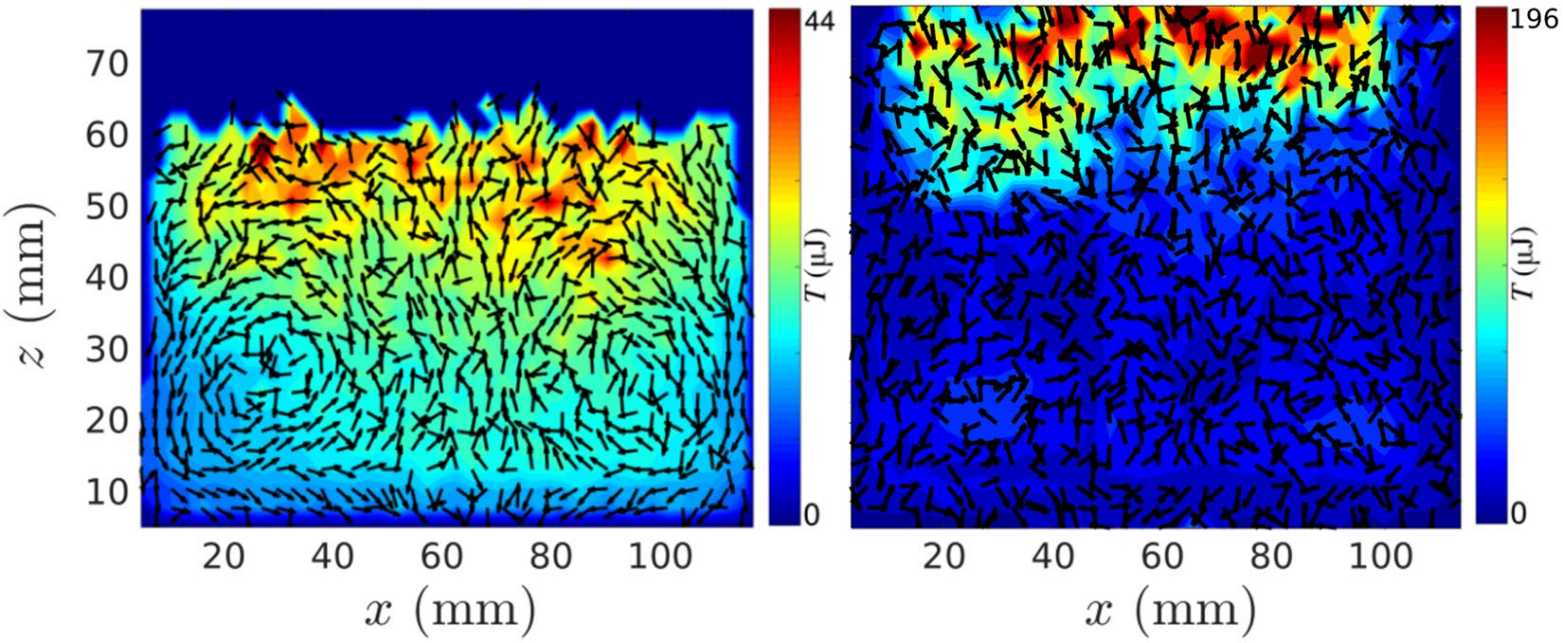
*Left:* Experimental velocity vector field showing ‘normal’ dual convection rolls for a bed of 5 mm nylon particles in a system with flat PMMA sidewalls. The field is superimposed on a two-dimensional granular temperature (*T*_*g*_) field, showing a tendency for particles to descend in the relatively low-*T*_*g*_, outer regions and ascend in the higher-*T*_*g*_ central region. *Right:* An otherwise identical system bounded by relatively elastic steel sidewalls showing seemingly random (i.e. non-convective) particle motion. Note that the images shown, and indeed all other similar plots shown in later figures, correspond to data that has been depth-averaged in the *y*-direction. This depth-averaging ensures that we observe only convective motion (or the lack thereof) in the plane of interest, with any bulk motion in the perpendicular *y*-*z* plane being averaged out.

In the presence of both a vertical and horizontal gradient in granular temperature^24,25^ (i.e. the mean local fluctuant energy of particles), particles descend in ‘cooler’ regions of the system and ascend in ‘hotter’ regions, giving rise to convective motion^16,17^. As such, it is possible to *deliberately induce*^16,17^ and *vary the strength*^26^ of convection by externally imposing such a gradient on a system. In past experiments, this has been achieved through the use of dissipative sidewalls, which act to lower the local temperature in their vicinity^5,16,17,26^. The left-hand panel of Fig. 1 shows an example of a pair of convection rolls created in such a manner. Indeed, although it has been shown^4^ that pseudo-thermal convection may, under suitable circumstances, occur spontaneously even in the absence of a (wall-induced) temperature gradient, the majority of prior experimental studies of pseudo-thermal convection rely on the dissipative nature of the vertical sidewalls bounding the system. Further, the vast majority, if not entirety, of prior works–be they experimental, theoretical or simulational–concern only simple, flat-walled systems. This limited study leaves many open questions. For example, under what specific conditions can convection be expected? Can it be induced (or inverted) via lateral energy *input* as opposed to *dissipation*? Is *directionality* of significance? And finally, on a more fundamental level, can we treat granular convection simply as a boundary-layer effect–i.e. if particle-particle restitution exceeds particle-wall restitution, downward-at-the-wall convection will inherently follow–or is the phenomenon more complex? If we are to truly understand this scientifically and industrially important phenomenon, we must address these crucial questions.

## Aims and Motivation

In this work, we explore a series of systems possessing differing arrangements of sawtooth-geometry walls; such asymmetric boundaries have, in other systems, been shown to induce directional motion via a ‘granular ratchet’ effect^27–29^. However, as our results demonstrate, such a geometry will also act to modify the local collision rate, and hence the system’s density and temperature distributions, thereby exerting a number of other effects which also strongly influence the dynamics of our system. Consequentially, we are able to provide first evidence of multiple novel mechanisms through which convective motion may be induced in dilute granular media, demonstrating that this convection is *not* inextricably linked to the presence of inelastic boundaries, nor even to the presence of gravity. We compare the relative strengths of each mechanism for a wide range of system parameters. In doing so, we notably observe that convection induced by dissipative sidewalls–the sole mechanism experimentally explored prior to this work–is in fact dominant only within a limited region of parameter-space. Finally, we demonstrate that the various mechanisms detailed allow us to not only induce or suppress convection, but also control its strength and even direction in a variety of manners.

## Methods

### Experimental System

**Figure 2:**
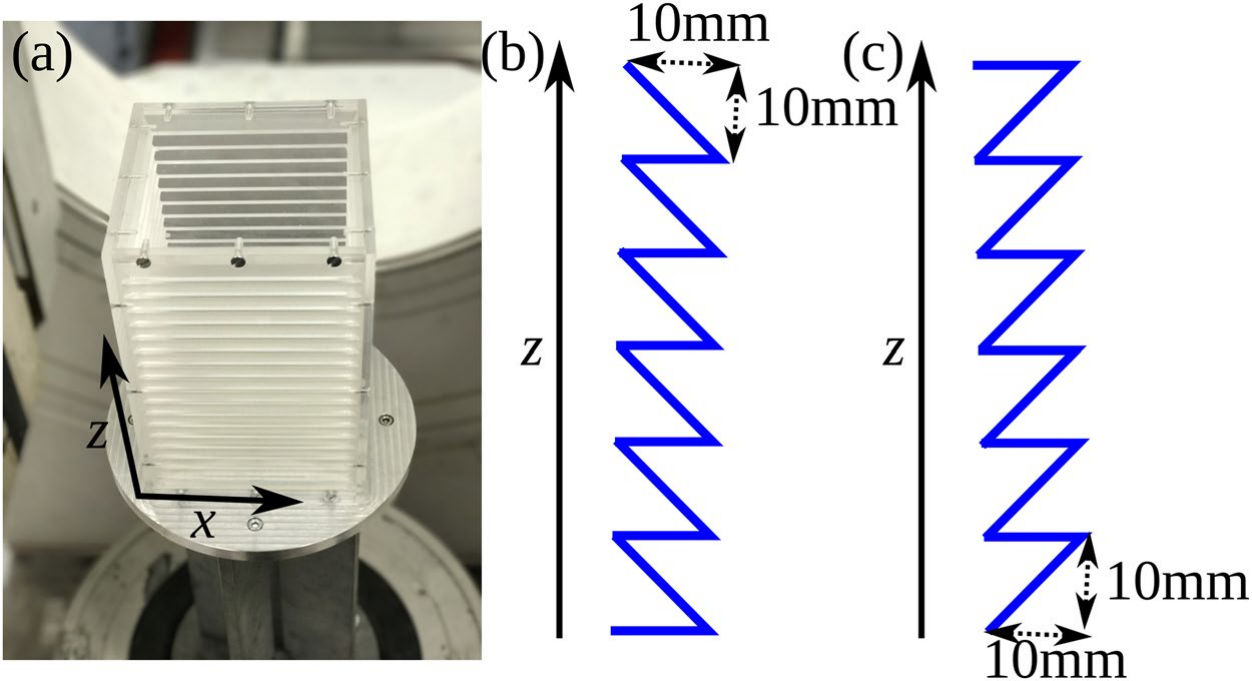
(**a**) Image of the experimental system showing the coordinate system used throughout this work. Images (**b**) and (**c**) show, schematically, the sidewalls’ ‘upward’ (inverse sawtooth) and ‘downward’ (regular sawtooth) configurations (see *Experimental Results*).

Our experimental system comprises a cuboidal cell of dimensions *L* × *W* × *H* = 120 × 120 × 250 mm affixed to an electromagnetic shaker and vibrated sinusoidally in the vertical (*z*) direction at constant frequency *f* = 75 Hz and amplitude *A* = 1.14 mm. The container houses a bed of *N* = 1250 (3350) 5 mm (3 mm) spherical Nylon particles, corresponding in each case to approximately two resting layers and providing, at the driving strengths used, a typical system density $\eta \sim 0.1$, representing a decidedly dilute (gaseous) regime; all experiments were repeated with both particle sizes. The strong, continual excitation to which the system is exposed causes the bed to exist in a dilute, fluid-like state^30^. The system is designed in a ‘modular’ manner, with interchangeable sidewalls enabling us to vary both the restitution coefficient, *ε*, of said boundaries, as well as their surface geometry. In the present work, we explore both a conventional ‘flat’ sidewall geometry as well as a sawtooth geometry intended to give directional impulse to the particulate systems excited (see Fig. 2).

In our introductory section, it was noted that differing convection mechanisms may occur in ‘dense’ and ‘dilute’ systems. In the granular community, however, there does not exist a single, widely-accepted criterion by which a discrete transition between the dense and dilute cases may be judged to exist. Rather, there exists a wide range of parameter space for which the frictionally-driven dense-convective mechanism and the dissipation-driven dilute-convective mechanism may be expected to coexist. Indeed, previous research^31^ has shown the pseudo-thermal mechanism to be active even in decidedly dense ($\eta  > 0.4$) systems. In spite of the presence of such a ‘crossover’ regime, it is nonetheless possible to ensure that the systems utilised here exist in an unequivocally dilute regime and hence exhibit *solely* pseudo-thermal convection, thus in turn ensuring that our results are clear and unambiguous in their interpretation. Specifically, we ensure that all systems from which data is extracted possess a characteristic coordination number $\langle Z\rangle  < 2$, meaning that particles are incapable of forming the stress-supporting chains necessary to the transmission of shear^32^, upon which the dense-convective convection mechanism is predicated^22,33^.

### Experimental Imaging–Positron Emission Particle Tracking

Positron Emission Particle Tracking (PEPT) is a non-invasive imaging technique which records, in all three spatial dimensions, the motion of a single, radioactively-labelled ‘tracer’ particle. PEPT utilises high-energy 511 keV gamma rays to track particles, facilitating the imaging of particles even deep within the interiors of dense, optically opaque systems with sub-millimetre accuracy and millisecond-scale temporal resolution^34^. We utilise nylon tracers, physically identical to all others within the system, which are activated via the adsorption of radioactive fluorine-18. Positrons emitted by the ^18^F rapidly annihilate with electrons in the tracer medium, producing pairs of *γ*-rays whose trajectories are separated by 180 ± 0.5°. By placing our experimental system between the dual heads of a positron camera, the straight-line trajectories of emitted *γ*-photon pairs may be reconstructed. By determining the intersection points of an adequately large number of such reconstructed paths, the position and thus–for a high enough activity and hence location rate–motion of the tracer may be reconstructed.

As the tracers used are (as mentioned above) identical to all others within the system and, in addition, systems such as those explored here may be safely assumed ergodic^16,35^, the long-time average of the motion of our single tracer particle may be used to extract a variety of one-, two- or three-dimensional fields corresponding to the behaviour of the system as a whole. In the present work, experimental data is acquired over a period of 3600 s, with an initial 900 s period being ignored in order to allow the system to reach a suitable steady state, and the remaining 2700 s of data being averaged over the in order to compute the various fields of interest (see Supplementary Material). While not crucial to the understanding of the current work, full details regarding the PEPT technique, including the manners in which the above-mentioned fields are determined from PEPT data, may be found in our Supplementary Materials and refs^34–36^.

### Discrete Element Method Simulation

In order to allow a deeper exploration of the systems studied, our experimental data is complemented by discrete element method (DEM) simulations performed using the *MercuryDPM* open-source software package^37–40^. The size, geometry and vibrational parameters of the modelled system are chosen to match experiment. Particles are modelled with a restitution coefficient *ε*_*p*_ = 0.9, density *ρ* = 1130 kgm^−3^ and frictional coefficient *μ* = 0.2. Walls are modelled with *μ* = 0.2 and various *ε* values. Preliminary tests varying *μ* show that, as in prior studies of dilute systems^26^,^41^, convective behaviour is largely invariant with the precise *μ* chosen–i.e. a small perturbation in the precise value of *μ* produces no qualitative change in the behaviour of the system. A similar observation was drawn also by Pontuale *et al*.^5^ in their recent study of wall-driven pseudo-thermal convection. Simulations of our full range of experimental data sets were found to produce, in all cases, consistent agreement in terms of the directionality and stability of the convection cells produced. Although not necessary to the comprehension of the current work, the interested reader may find additional details of our simulations in our Supplementary Materials.

The two-dimensional velocity, temperature and density fields (both experimental and numerical) presented throughout this manuscript are depth-averaged in the *y*-direction, thereby ensuring both that the data presented are representative of the full system, and that the influence of any bulk motion in the *y*-*z* plane on the data displayed in our plane of interest is minimized.

## Results and Discussion

### Experimental Results

As discussed previously, it is well known^5,26,42–44^ that cuboidal systems with flat, dissipative sidewalls may produce dual convection rolls with particles flowing downward near the edges and upward in the centre of the system. For a system of relatively elastic nylon particles (*ε* ≈ 0.9^45^) bounded by comparatively inelastic^16^ PMMA sidewalls, we are able to recreate the expected results (see Fig. 1, left-hand panel). Replacing the PMMA sidewalls with more elastic steel (*ε* ≈ 0.9^46^) sidewalls, meanwhile, leads to a suppression of convective motion (see Fig. 1, right-hand panel) due to the effective elimination of the horizontal temperature gradient present in the dissipative-walled case, once again in-line with prior expectations^5,26,42^. Having established that the system behaves as expected for known cases, we investigate next previously unexplored cases in which we vary not the sidewall *material* but their *geometry*. In the first case explored, the system is bounded by four PMMA sidewalls with an inverse sawtooth pattern (see Fig. 2). The oscillating walls can be expected to create a net impulse whose direction is determined by the sawtooth orientation^28^.

**Figure 3:**
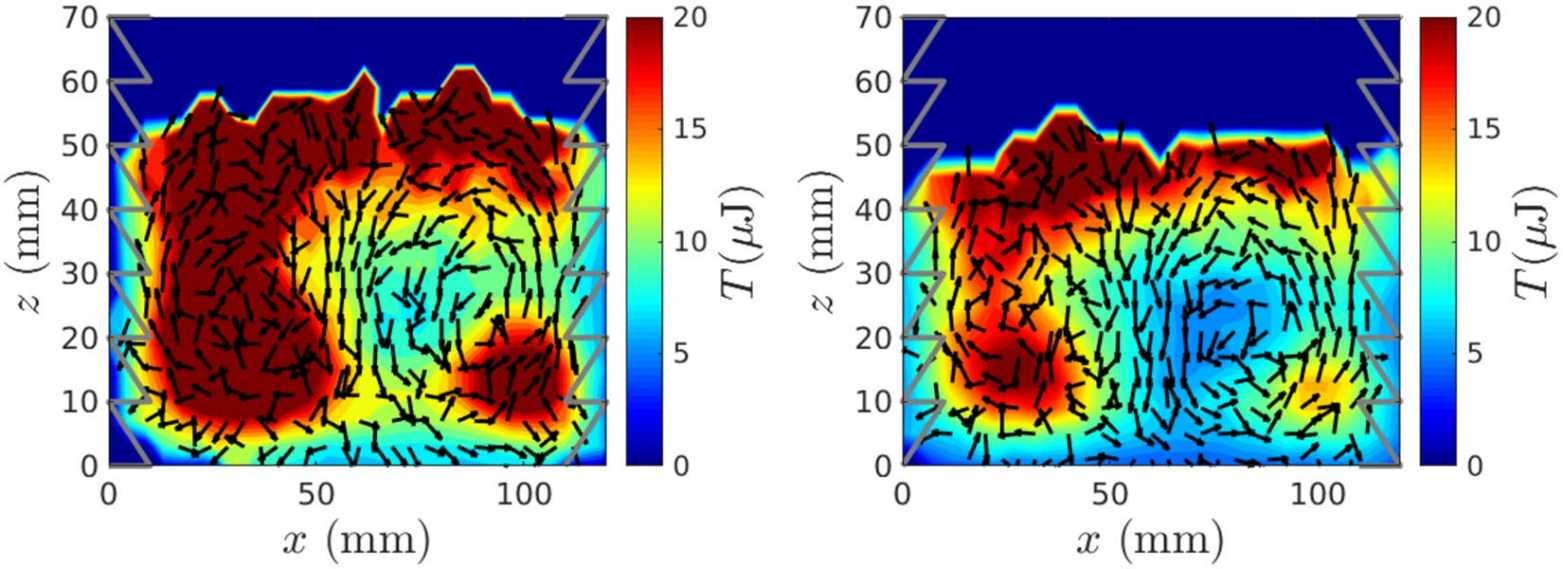
Experimental velocity vector fields showing inverse convection rolls for identical beds of 5 mm nylon particles housed in systems whose walls possess exclusively ‘upward’ (*left*) and ‘downward’ (*right*) sawtooth geometries. As in Fig. 1, the velocity fields are superimposed on a granular temperature field, showing particles to ascend in the relatively ‘hot’ outer regions and descend in lower-*T* central regions–irrespective of the sawtooth orientation, and hence the direction of impulse provided to colliding particles. Grey lines superimposed on the images represent the basal positions of the sawtooth walls.

With the system in the configuration (Fig. 2(b)) we again observe a two-roll convection pattern, but this time with particles travelling *upward* at the lateral walls and *downward* near the system centre (see Fig. 3(a)). While the re-orientation of convection in dilute systems has previously been observed in experiment by Pontuale *et al*.^44^ through the use of a container with canted sidewalls, this is the first time that such convective motion has been observed in a system bound by ostensibly vertical sidewalls. The form of the re-oriented rolls also differs significantly between the current work and that of Pontuale *et al*.^44^: in the prior work, the inclination of the walls changes the form of the convection observed entirely, breaking the symmetry of the ‘normally-convective’ case and forming a single roll travelling upward at one boundary and downward at the other; in the present case, the symmetric two-roll flow is maintained, with only the sense of the flow of said rolls changing. Note that our observed inverse convection is observed despite the continued use of the *comparatively dissipative* PMMA walls, providing direct evidence that wall-driven granular convection is *not* inherently contingent upon the elastic properties of a system.

However, before we ascribe this observed behaviour to a granular-ratchet-like effect, we must consider also the case in which the sawtooth direction is inverted (Fig. 2(c)). Interestingly, in this scenario, where the walls may be expected to provide a net *downward impulse*, the direction of convection *remains upward* at the wall (see Fig. 3). This is a particularly illuminating result, as it demonstrates particles to still move upwards in the higher-*T*_*g*_ region of the system even for a case in which the net impulse provided to the system *and* the comparatively dissipative sidewall would *both* be expected to create downward motion in the vicinity of the horizontal boundaries. In other words, the convective instability is initiated by the locally increased (scalar) temperature, rather than being prescribed by the *net direction* of energy input. To put it differently, pseudo-thermal convection in a granular system is *not* a *boundary-layer* effect, but rather a *collective effect*.

An interesting additional feature of Fig. 3 is the apparent decrease in the *T*_*g*_ in the immediate vicinity of the vibrating sawteeth, both in the upward- and downward-oriented cases. While this observation may immediately seem contradictory to our central thesis, it can in fact be relatively easily explained. Specifically, particles undergoing collisions with said oscillating sawteeth will possess a higher (kinetic) *energy* than particles further from the wall. However, the velocities of these particles will also become more strongly correlated, leading to a reduction in the mean local *fluctuant energy*, i.e. a lower granular temperature. As such, the peak in *temperature* can be expected to occur in the vicinity of the inner-edges of the sawteeth, where this high energy is randomised and redistributed through particle-particle collisions–as is indeed observed in Fig. 3. Pleasingly, this observation further supports the concept of pseudo-thermal convection as a collective (as opposed to boundary) effect.

**Figure 4:**
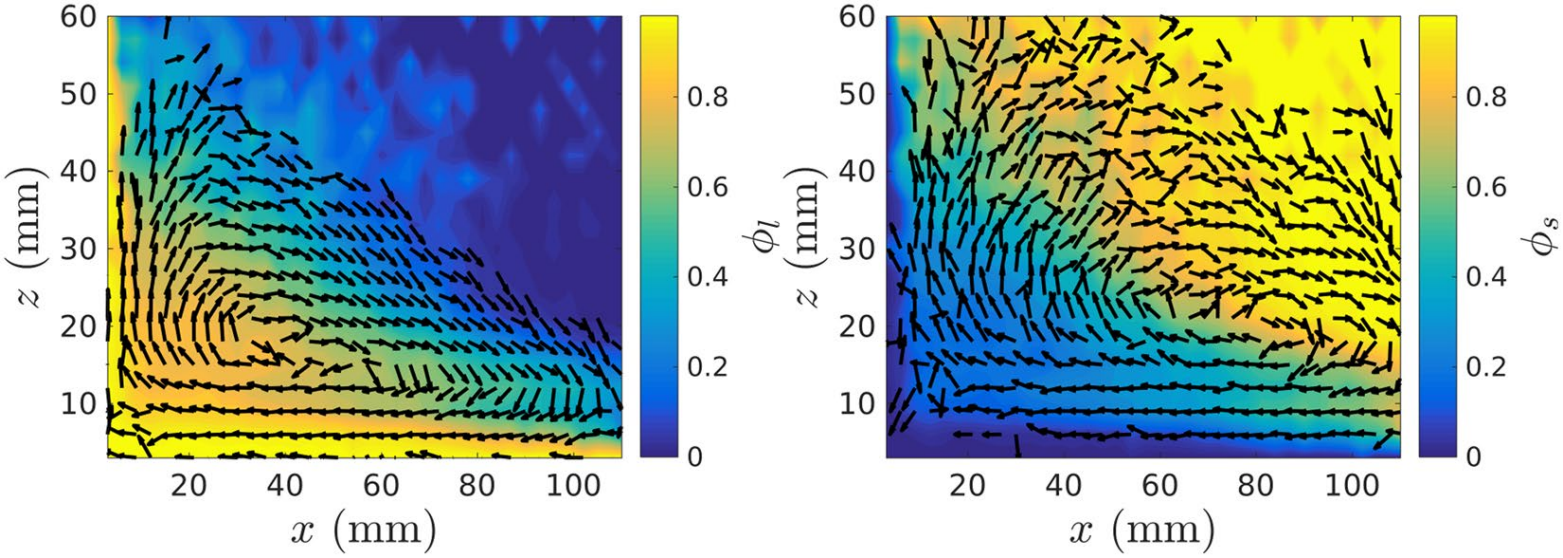
Velocity vector fields showing convection patterns for the larger (*left*) and smaller (*right*) components of a bidisperse-by-size granular bed of 3 and 5 mm particles. The bed is bounded in the *y*-direction by a pair of flat steel walls and in the *x*-direction by a single flat, PMMA at the right (*x* = 120 mm) boundary and an upward-facing sawtooth wall at the left-hand (*x* = 0 mm) boundary. The colouring of the figure depicts the spatial variation of the local relative volume fractions of large $({\varphi }_{l}=\frac{{\eta }_{l}}{{\eta }_{l}+{\eta }_{s}})$ and small $({\varphi }_{s}=\frac{{\eta }_{s}}{{\eta }_{l}+{\eta }_{s}})$ particles, with *η*_*χ*_ here representing the local packing density of species *χ*.

Our experimental results also demonstrate that, by utilising differing combinations of flat and sawtooth wall geometries, the convection patterns within the system may be deliberately altered. For example, a system comprising two adjacent sawtooth and two adjacent flat walls is observed to create only a *single* convection roll, oriented along the diagonal of the system. Similarly, a system with a pair of opposing flat steel walls bounding the *y*-direction and, in the *x*-direction, an upward facing sawtooth wall at *x* = 0 and a flat PMMA wall at *x* = 120 mm will produce a single roll in the *x*-*z* plane (see, for example Fig. 4). While this observation is not, considering the preceding discussions, overly surprising, it nonetheless exhibits the more practical utility of the results presented here, demonstrating that we can *deliberately control* the convective behaviours of a system–including the number, orientation and sense of the rolls produced–in a manner not previously observed.

Figure 4 also demonstrates that our observations for monodisperse systems hold also for the binary case, with both species exhibiting the qualitative convection patterns expected from the previously-discussed unary systems, but larger and smaller particles segregating, respectively, towards the lower-left and upper-right regions of the system. It is interesting to note that this observed segregation is in stark contrast to the well-known ‘Brazil-Nut Effect’^47^, whereby larger particles segregate directly upward through a given granular bed. While external to the focus of the current work, the complex dynamics and segregation patterns observed in the binary systems tested will be discussed in detail in a future publication.

### Simulation Results

In order to cross-validate our experiments and simulations, the systems detailed in the preceding sections were reproduced in simulation. Our numerical results were found to produce, in all cases, consistent agreement in terms of the directionality and stability of the convection cells produced, providing a significant measure of support for the validity of our simulations (see Supplementary Material section S2.1). Having been validated against experimental results, our simulations allow us to investigate a far broader range of systems and regions of parameter space, including those not realisable experimentally.

**Figure 5:**
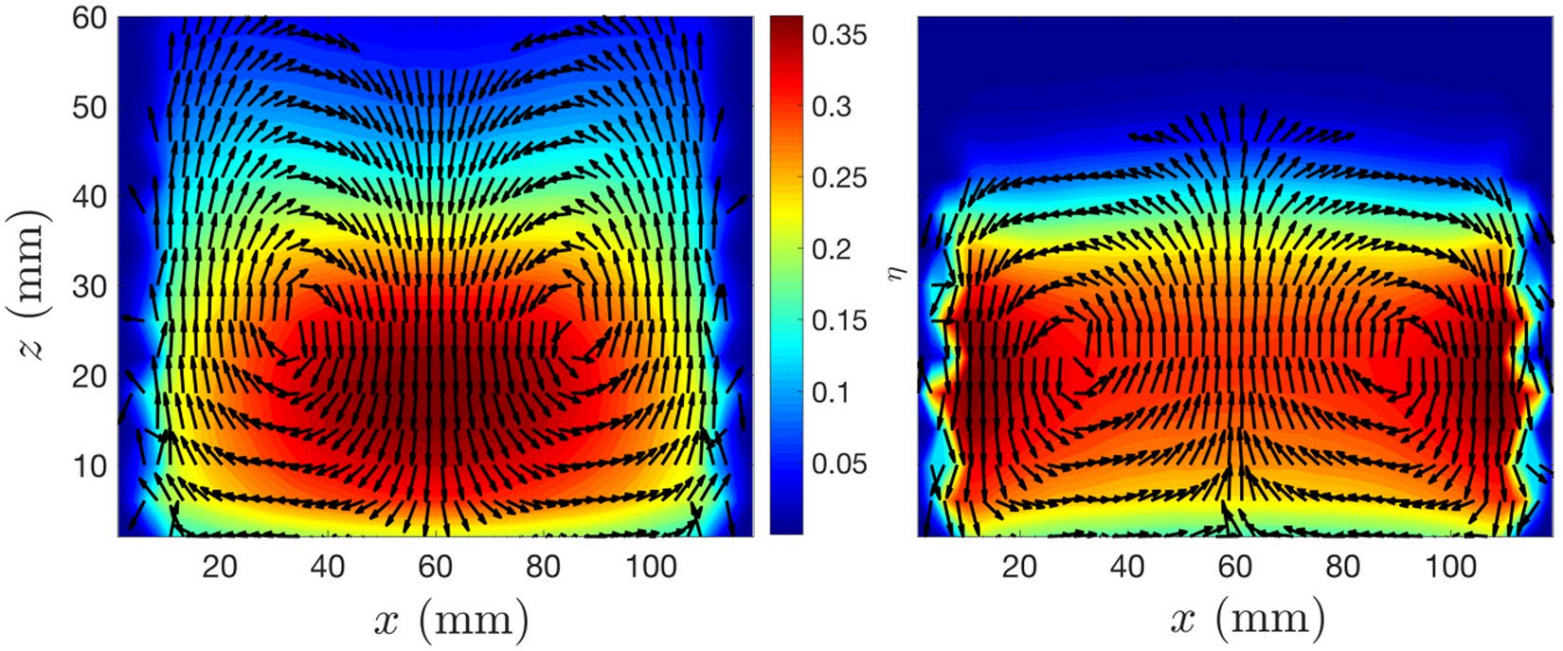
Simulated velocity vector fields for systems with vertically vibrating (*left*) and static (*right*) inverse-sawtooth sidewalls superimposed on their respective depth-averaged packing density distributions. In both cases, *ε*_*p*_ = *ε*_*w*_ = 0.9. All other parameters correspond to experimental values. Note that in both cases particles ascend (descend) in regions of lower (higher) packing density, *η*.

Firstly, a series of simulations were conducted for a vibrating base but *static* sawtooth walls for a variety of sidewall restitution coefficients (*ε*_*w*_). Remarkably, for both the sawtooth and inverse sawtooth configuration and for *all ε*_*w*_ values tested–including the case of *perfectly elastic* walls (*ε*_*w*_ = 1)–exclusively ‘conventional’ (down at walls, up at centre) convection was observed. This is a valuable result, as it demonstrates that a ratio $\frac{{e}_{w}}{{e}_{p}} < 1$ is *not requisite* for the induction of dissipation-driven convection. Our observations can be relatively easily explained: in this case, the localised decrease in *T*_*g*_ near the walls is caused not by the dissipative properties of the walls themselves, but rather by an increased local inter-particle collision rate induced by the sawtooth geometry which, in turn, leads to increased dissipation and thus a reduced *T*_*g*_^48^. In other words, the direction of convection is determined solely by the net energy flux in the vicinity of the boundary, and *not* the specific elastic properties of the wall. Figure 5 compares a pair of otherwise identical systems with moving (*left*) and static (*right*) walls, clearly showing the expected increased particle density near the walls for the static case. The above represents a remarkable observation, and one which provides further support for our conjecture that pseudo-thermal convection is indeed a collective effect.

**Figure 6:**
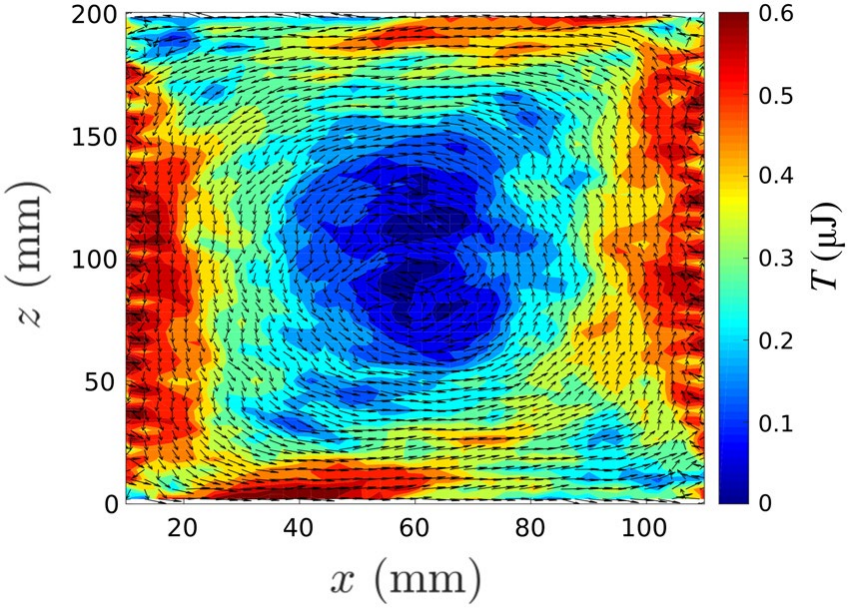
‘Ratchet-driven’ convection for a system with vertically-oscillating sawtooth sidewalls oriented in the downward-facing configuration (Fig. 2(c)) at the system’s left-hand wall (*x* = 0 mm) and the upward-facing configuration (Fig. 2(b)) at the system’s right-hand wall (*x* = 120 mm) in the absence of gravity and with a static (i.e. non-vibrating) base.

Lastly, a series of simulations were conducted in zero gravity. In the absence of an external field, we observe (comparatively weak) convective motion whose sense is determined by the sawtooth orientation (see Fig. 6) travelling upward at an upward-facing oscillating sawtooth wall placed at the system’s right-hand boundary, and downward at the downward-facing oscillating wall at the opposing boundary. In other words, when not drowned out by more dominant mechanisms, our system will indeed exhibit a granular-ratchet-like effect. Nonetheless, the introduction of even a weak ($\tilde{g}=\frac{g}{10}$) gravitational field is sufficient to re-introduce the inverse (upward at both boundaries) convection described in the preceding section, implying that the ratchet-driven motion is present only in the absence of a convection-driving heat flux. This clear demonstration that the ratchet-driven mechanism–which acts only on a single layer of particles–is decidedly sub-dominant to the influence of temperature once again reaffirms the nature of pseudo-thermal convection as a collective effect.

### Comparing segregation mechanisms

Having demonstrated the existence of several novel methods of inducing convection, we now compare and contrast the relevant mechanisms underlying each and their relative importance under differing conditions. Having already established the weak nature of the above-described ratchet-driven mechanism, we focus here on the remaining three, where the convective instability is introduced via localised cooling due to wall inelasticity (*Mechanism 1*), localised cooling due to sidewall geometry (*Mechanism 2*) and localised heating from a moving sawtooth wall (*Mechanism 3*). Though both *Mechanism 1* and *Mechanism 2* provide cooling in the vicinity of the system’s lateral boundaries, the former arises predominantly due to direct *particle-wall* collisions (making *ε*_*w*_ the relevant control parameter), while *Mechanism 2* is predicated on an enhanced local (i.e. ‘near wall’) *particle-particle* collision frequency (making *ε*_*p*_ and the more relevant parameter).

We firstly compare mechanisms *1 & 2* via a series of simulations varying both the inter-particle (*ε*_*p*_) and particle-wall (*ε*_*w*_) restitution coefficients in the range *ε*_*p*,*w*_ ∈ [0, 1], under the constraint ${\varepsilon }_{p} < {\varepsilon }_{w}$, meaning that if *Mechanism 2* is dominant, coventional wall-driven convection will be observed, whereas if *Mechanism 1* dominates, we expect inverse convection. Although a full range of *ε* values were tested, buoyancy-driven convection was, naturally, only observed for a subset of this parameter space: for ${\varepsilon }_{p}\lesssim 0.75$, the high dissipation and resultant dense packing prevents full pseudo-thermal convection for all *ε*_*w*_. Conversely, as ${\varepsilon }_{p}\cdot {\varepsilon }_{w}\to 1$, the system simply exhibits chaotic motion, as may be expected^49^.

Remarkably, for *all* (convective) cases, rolls were oriented *downwards* at the walls–even for cases in which *ε*_*w*_ is *markedly higher* than *ε*_*p*_. In other words, for the entire parameter space tested, *Mechanism 2* is *decidedly dominant* over *Mechanism 1*. While this observation cannot be assumed to be universal, the consistent dominance of *Mechanism 2* observed for the physically realistic parameters explored here indicates that convective motion may be better controlled by varying wall *geometry* as opposed to wall *material* (the property studied in all prior experimental works). This is a potentially highly-useful observation both for future researchers wishing to further investigate the convective phenomenon, as well as for industry, where the ability to induce homogeneity in particulate systems via convective remixing is widely desirable^8,50,51^.

While a full theoretical treatment concerning the above observation is beyond the scope of the current work, a brief, first-order explanation of this observation may nonetheless be proffered. Consider a system possessing two separate regions, *A* and *B*, where the mean fractional energy loss per collision can be estimated as $(1-{\varepsilon }_{i}^{2})$ and a number *n*_*i*_ (where *i* = *A*, *B*) collisions occur per unit time. The dissipation in region *i* is therefore proportional to ${D}_{i}=(1-{\varepsilon }_{i}^{2{n}_{i}})$. Based on our knowledge of convection we may assume, to first order, that if ${D}_{A} > {D}_{B}$, particles will descend in region *A* and ascend in region *B*, and vice-versa. The above inequality can be re-written as:

1 $$\frac{{n}_{A}}{{n}_{B}} > \frac{\mathrm{ln}\,{\varepsilon }_{B}}{\mathrm{ln}\,{\varepsilon }_{A}}$$

In other words, if we design our geometry such that particles encounter on average twice as many collisions per unit time in the (wall) region *A* as in the (centre) region *B*, then even for relatively elastic (*ε*_*w*_ = 0.9) walls, the *maximal* mean particle-particle restitution coefficient for which net downward motion may be observed in region *B* (i.e. *Mechanism 1* drives convection) is 0.81. For the convective region of the phase space explored, the typical ratio of collision rates lies in the range $3.5\lesssim \frac{{n}_{A}}{{n}_{B}}\lesssim 7$. Taking even the lower bound of this range, the upper limit for *ε*_*p*_ falls to 0.69, well below the convective threshold noted above. While this simple theory is divergent as *ε*_*w*_ → ∞ and hence cannot be applied for the case of perfectly or near-perfectly elastic walls, it nonetheless provides a measure of explanation for the prevalence of *Mechanism 2* in our simulations.

**Figure 7:**
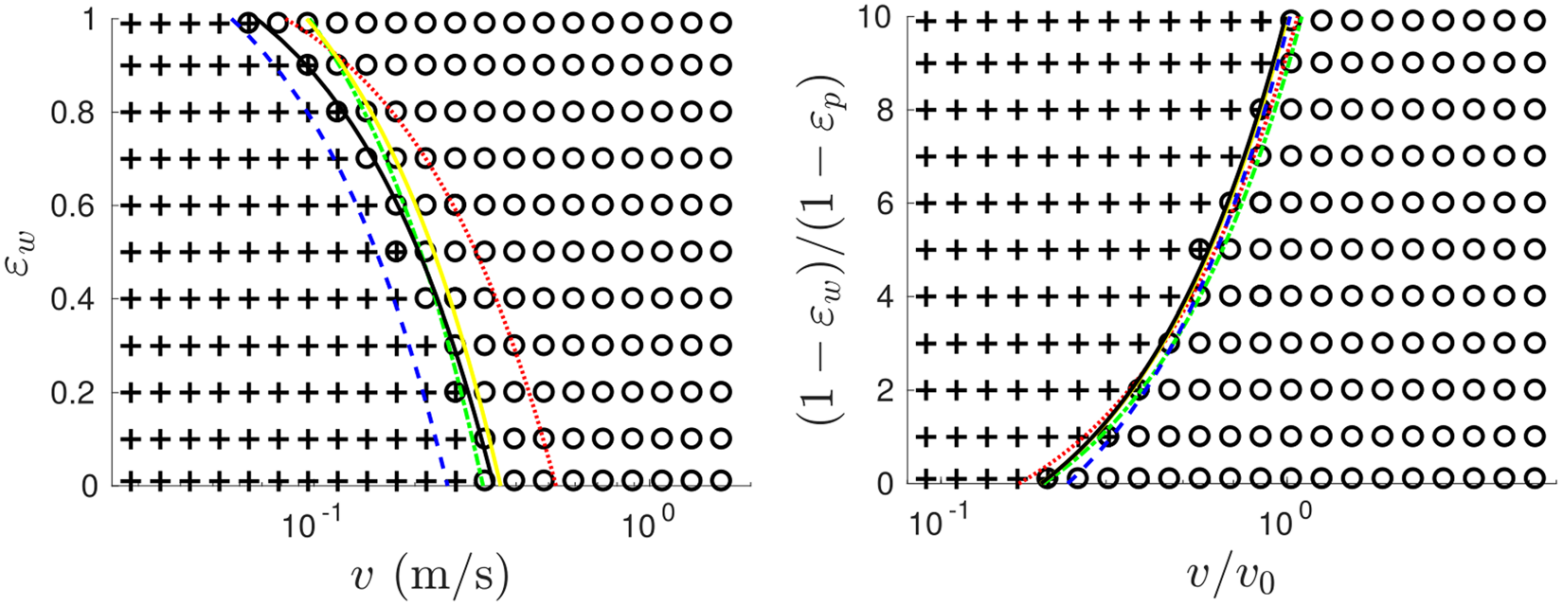
Original (*left*) and normalised (*right*) phase diagrams showing the transition from ‘conventional’ (crosses) to ‘inverse’ (circles) convection for *v*_0_ = 0.3468 m/s, *ε*_*p*_ = 0.9 (symbols and solid black line). Metastable states in which both conventional and inverse rolls are observed are denoted by crosses superimposed on circles. Shown also are the phase boundaries for the cases of doubled and halved base energy input, i.e. ${v}_{0^{\prime} }=\sqrt{2}{v}_{0}$ (red dotted line) and ${v^{\prime} }_{0}=\frac{{v}_{0}}{\sqrt{2}}$ (blue dashed line), and for differing particle elasticities *ε*_*p*_ = 0.85 (solid yellow line) and *ε*_*p*_ = 0.8 (green dot-dash line), both driven with the increased velocity $\sqrt{2}{v}_{0}$.

Finally we consider *Mechanism 3*. In simulation, we may vary the vibrational velocities, *v* = 2*πfA*, of the system’s sawtooth walls (*v*) and base (*v*_0_) *individually*, allowing us to independently vary sidewall energy-injection and total energy input. Figure 7 presents, for various system energies and particle restitution values (*ε*_*p*_), a phase diagram in *ε*_*w*_-*v* space showing the transition boundary from conventionally-oriented convection (where the combined effects of *Mechanisms 1, 2* dominate) to inverse convection (*Mechanism 3* dominates). The phase boundaries for various *v* and *ε*_*p*_ values, when correctly normalised, are observed to collapse, suggesting a universal–or at least broadly applicable–phase boundary for our given system geometry. The boundary is well-described by a linear function whose approximate form can be empirically determined as:

2 $$\frac{1-{\varepsilon }_{w}}{1-{\varepsilon }_{p}}=\alpha \frac{v}{{v}_{0}}-\,\beta $$

where, for the current system, *α* = 12 ± 1 and *β* = 2.4 ± 0.4. The observed linear form makes physical sense as, for example, doubling the value of *v* can be expected to approximately double the mean velocity imparted by the walls^52^, while doubling (1 − *ε*_*w*_) will–to a first approximation–double the rate at which velocity is lost to the walls–i.e. one may indeed expect a system at [*kv*, *k*(1 − *ε*_*w*_)] to behave approximately equivalently to a system at [*k*,(1 − *ε*_*w*_)]. The negative intercept also agrees with our observations that a finite *v* is required in order to induce inverse convection. Finally, the dependence on the ratio $\frac{v}{{v}_{0}}$ also aligns with physical expectations, as the direction of convection within the system is determined by the direction of the energy flux at the walls, determined by the ratio of the mean pseudo-thermal velocity of particles within the system (which in turn depends on *v*_0_) to the velocity imparted by the oscillating walls. While the precise values of *α* and *β* will, naturally, vary with the properties of the system and particles in question, the simple, linear nature of our equation (2) suggests that, with only two known data points, one may predict the full phase boundary for any given system of interest, and hence the convective state of said system for any arbitrary combination of *v*, *v*_0_, *ε*_*p*_ and *ε*_*w*_.

### Interplay with Bulk Buoyancy-Driven Convection

Due to the large parameter space explored, many of our systems will exist in or near the region of the inelasticity/gravity/driving-strength parameter space where ‘bulk buoyancy-driven’ (BBD) convection^5,18,53^ may, due to the innate instability of the non-convective state under such conditions, be expected to occur spontaneouslty, even in the absence of dissipative boundaries. As such, we must consider how we can reconcile this ‘classical’ bulk mechanism with the various wall-driven mechanisms proposed here. In fact, the two may be relatively easily reconciled by considering the new mechanisms presented here as both extensions to, and special cases of, BBD convection. In order to illustrate the above point, let us consider two distinct cases, respectively outside and inside the range of parameter space for which BBD convection would normally be expected. In the first case–outside the classical BBD regime–our various mechanisms may be seen as inducing buoyancy-related convective motion which would not otherwise (spontaneously) occur, thus in essence acting to extend buoyancy-driven (pseudo-thermal) convection beyond the normal BBD parameter space. In the second case–within the classical BBD regime–though convection may be expected to occur even in the absence of a horizontal temperature gradient, the presence of energy-inducing or energy-removing boundaries will inherently provide the initial instability required to initialise convection, and hence determine the location and orientation of convection rolls. In other words, instead of a random convection pattern forming due to a random instability (as in the case of classical BBD systems with no imposed temperature or density gradients) here the system is ‘forced’ to adopt a specific form, geometry, orientation and sense according to the system’s boundary properties. To put it concisely, in this latter case our mechanisms can be seen as acting as both a ‘trigger’ and ‘anchor’ for BBD convection.

The interesting natural conclusion of the above is that we can expect the behaviour of our system, in terms of the form, location and directionality of the convective flow patterns formed, to be effectively agnostic of its position within or outside of the classical BBD parameter space–i.e. said directionality can be predicted solely through consideration of the mechanisms described within this article.

### Generality of Results

**Figure 8:**
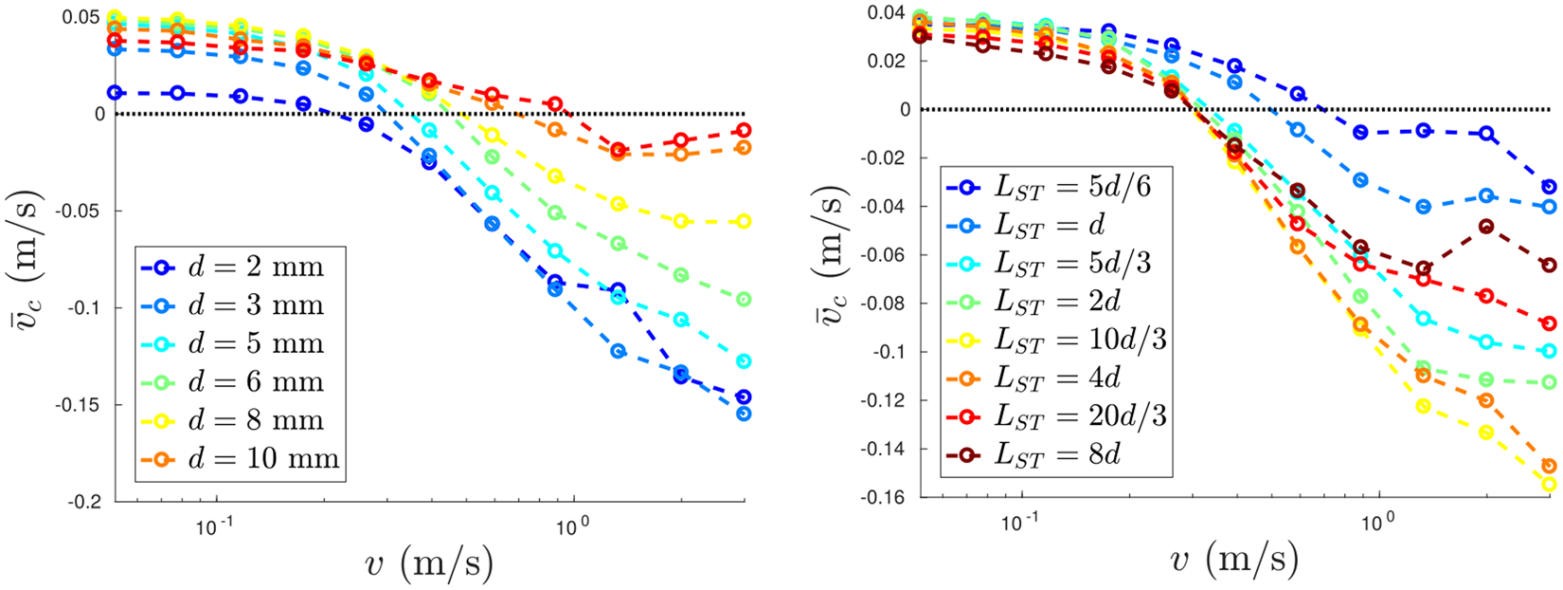
Mean convection velocity, ${\bar{v}}_{c}$, as a function of the sidewall oscillation velocity, *v*, at fixed base velocity *v*_0_ = 0.52 m/s and particle-particle and particle-wall restitution coefficients *ε*_*p*_ = 0.9 and *ε*_*w*_ = 0.5, respectively. Data is shown for various particle sizes, *d*, at fixed particle volume (*left*) and differing sawtooth sizes, *L*_*ST*_, at fixed *d* (*right*). In all cases, positive values of ${\bar{v}}_{c}$ correspond to normal convection, and negative values to inverse convection. The dotted black line at ${\bar{v}}_{c}=0$ is shown as a guide to the eye, highlighting the positions of the transitions between the two convective states for the various data sets shown.

In the preceding sections we have expounded a number of interesting properties of, and phenomena relating to, vibrated granular systems possessing sawtooth-geometry walls. However, the results presented thus far pertain exclusively to the case of a single sawtooth size, and two distinct but not strongly dissimilar particle diameters. As such, it is important to establish whether our results are specific to the precise cases explored, or whether they are in fact generalisable to a broader range of systems. In the left-hand panel of Fig. 8 we show the mean convection velocity, ${\bar{v}}_{c}$, of a simulated (upward-oriented) sawtooth-walled system as a function of the wall oscillation velocity, *v*, at fixed base velocity *v*_0_ = 0.52 m/s for various particle diameters *d* ∈ [2, 10] at a fixed particle volume equal to that used in our main experiments. Firstly, and most importantly, we note that the system is capable of producing both conventional and inverse convection for the full range of particle sizes explored, including for the case in which the particle diameter, *d*, is equal to the sawtooth width, *L*_*ST*_. However, the data also clearly shows a shift in the transition point between the two convective regimes, as well as a strong variation in the strength of convection, as *d* is varied. Specifically, we find that larger particles typically exhibit a later transition to the inverse-convective regime (i.e. require a higher wall-energy in order for convection to be reoriented), and show a generally weaker (inverse) convection rate–though the apparent relation between particle size and the strength of conventional convection is less clear-cut.

However, it must be remembered that in varying *d* at fixed system volume we are not only varying the particle size, but also the effective width of the bed and, by definition, altering the number, *N*, of particles within the system, which in turn may strongly influence the energy dissipation rate^54^ and hence mean packing density^55,56^ of the system–all of which may influence the convective behaviours of a granular fluid^16,26,57,58^. In other words, the trends observed in the left-hand panel of Fig. 8 cannot be ascribed solely to the effects of particle size, or rather the more relevant dimensionless parameter $\frac{{L}_{ST}}{d}$, which represents the ratio of sawtooth size to particle size. As such, additional simulations were performed at a fixed particle size *d* = 3 mm and with varying *L*_*ST*_ (Fig. 8, right-hand panel). From these additional data, we can gain various new insights into the behaviours of our system. Firstly, we observe that for the normally-convective case, ${\bar{v}}_{c}$ is typically higher for systems with smaller sawteeth, i.e. a smaller ratio $\frac{{L}_{ST}}{d}$. Such an observation makes physical sense, since as $\frac{{L}_{ST}}{d}\to 0$ the system will approach the case of a flat-walled geometry. In other words, as the relative size of sawtooth to particle decreases, the net energy input rate will also decrease, meaning that energy *dissipation* at the walls becomes increasingly dominant, thus facilitating stronger *normal* (i.e. dissipation-driven) convection.

A second notable feature is that a similar transition point from normal to inverse convection is observed for all $\frac{{L}_{ST}}{d} > 1$. This is a pleasing result, as it implies that the phase diagrams of Fig. 7 and equation (2) may be expected to hold, to a reasonable degree of accuracy, for any convective system for which $\frac{{L}_{ST}}{d} > 1$. Indeed, the data show that the transition points for all cases fulfilling this inequality fall within the range [0.26,0.38], which compares favourably with the predictions of equation (2) which, considering the quoted margins of error, predict a transition point lying in the range [0.28,0.37]. Note here our deliberate use of the phrase *convective system*, as for $\frac{{L}_{ST}}{d}\gg 1$ the two-roll convective flow regime of our system is observed to become unstable. Conversely, as the normalised sawtooth size reaches unity, we note a sharp and significant jump in the transition point, and a notable weakening of the convection strength observed in the inverse-convective regime. The elucidation of such a ‘minimum effective sawtooth size’ may prove valuable to future researchers hoping to explore similar systems.

Finally, in the inverse-convective regime, we observe a non-monotonic variation in convection strength (${\bar{v}}_{c}$) with $\frac{{L}_{ST}}{d}$, implying that there exists an optimal sawtooth size for inducing strong inverse convection. The determination of this optimal value, and its variation with key system parameters, will form the focus of a future publication.

## Conclusions

Through exploration of a particulate assembly bound by vertical sidewalls possessing variable geometric and elastic properties, we have provided significant new insight into the phenomenon of pseudo-thermal granular convection, demonstrating several distinct, novel manners in which convective motion may be induced, and elucidating the fundamental physics underlying said motion. We have presented first experimental evidence of ‘inverse’ wall-driven convection in dilute granular systems bounded by vertical walls and, further, determined a simple empirical form for the boundary delineating normally- and inversely-convective states, which may prove a useful predictive tool for future researchers.

In addition, we have compared the relative strengths of the various mechanisms observed showing, somewhat surprisingly, that the only previously-documented mechanism is in fact *decidedly sub-dominant* across a broad range of multi-dimensional parameter-space. We found also that while directional motion imposed by a ‘ratchet effect’ may induce circulatory motion within the system, such motion is readily overcome by other convective mechanisms in the presence of a gravitational field and the resultant vertical temperature gradients and energy fluxes. Importantly, our results have demonstrated a number of manners in which we may deliberately alter the strength, orientation, directionality and number of convection rolls in a system, an observation carrying clear potential for industrial and scientific applications, and opening the door for significant further study.

Finally, and perhaps most significantly, we have provided new, fundamental insight into the nature of pseudo-thermal convection, showing that boundary-layer effects (e.g. directional motion imposed by walls possessing asymmetric geometry) exert only a secondary influence on the behaviour of our systems. Rather, convective motion within a dilute granular system is a collective phenomenon, its strength and direction determined by the local energy fluxes near the boundaries of the system, as opposed to the specific physical properties of these boundaries alone.

## Electronic supplementary material

Supplementary Material

## Data Availability

The datasets generated during and/or analysed during the current study, and the codes used for the generation and analysis thereof, are available from the corresponding author on reasonable request.
