# Supplementary material for: New Insight into Pseudo-Thermal Convection in Vibrofluidised Granular Systems
Source: Sci Rep. 2018 Aug 27;8:12859. doi: 10.1038/s41598-018-31152-8 (PMC6110765; doi:10.1038/s41598-018-31152-8)
Supplement: Supplementary file 1 — Supplementary Material [file 41598_2018_31152_MOESM1_ESM.pdf]

New Insight into Pseudo-Thermal Convection in Vibrofluidised Granular Systems: Supplementary Material

C. R. K. Windows-Yule[1,2,*], E. Lanchester[2,+], D. Madkins[2,+], and D. J. Parker[2]

[1]Institute for Multi-Scale Simulation, Friedrich-Alexander Universität Erlangen-Nürnberg, Schloßplatz 4, 91054 Erlangen
[2]School of Physics and Astronomy, The University of Birmingham, Edgbaston, Birmingham, B15 2TT, UK
[*]windowsyule@gmail.com
[+]these authors contributed equally to this work

1 S1: Positron Emission Particle Tracking

Positron Emission Particle Tracking (PEPT) is a non-invasive imaging technique which records, in all three spatial dimensions, the motion of a single, radioactively-labelled 'tracer' particle. As PEPT utilises high-energy 511 keV gamma rays to track particles, it enables the imaging of particles even deep within the interiors of dense, optically opaque systems with sub-millimetre accuracy and millisecond-scale temporal resolution[1].

In the present work, we utilise nylon tracers, physically identical to all others within the system, which are activated via the adsorption of radioactive fluorine-18. Positrons emitted by the fluorine 18 rapidly annihilate with electrons in the tracer medium, producing pairs of γ-rays whose trajectories are separated by $180 \pm 0.5°$. By placing our experimental system between the dual heads of a positron camera, the straight-line trajectories of pairs of emitted gamma photons may be reconstructed.

By determining the intersection points of an adequately large number of such reconstructed paths, the position and thus – for a high enough activity and hence location rate – motion of the tracer may be reconstructed.

As the tracers used are (as mentioned above) identical to all others within the system and, in addition, systems such as those explored here may be safely assumed ergodic[2,3], the long-time average of the motion of our single tracer particle may be used to extract a variety of one-, two- or three-dimensional fields corresponding to the behaviour of the system as a whole.

Below, we describe in detail the manners in which the fields most relevant to the current study may be extracted from PEPT data.

1.1 S1.1: Particle Density Fields

In order to determine a two-dimensional density field for our system, we begin by subdividing the experimental volume into a series of equally-sized 'cells' in the plane of interest. By measuring the time spent by the tracer within each cell and normalising by the total duration of the experiment, we may determine a local 'residence fraction', F_R, for each given region of the system.

For an ergodic, steady-state system, the mean particle density within any given cell may be assumed to be directly proportional to F_R, allowing us to determine the number density, n, within the ith cell of the system as:

$$n^i = \frac{NF_R^i}{V_c} \tag{1}$$

where N is the total number of particles in the system as a whole and V_c the cell volume. With a known particle diameter, d, the local packing density can then be determined as:

$$\eta^i = n^i \frac{\pi d^3}{6} \tag{2}$$

1.2 S1.2: Velocity Fields

In order to visualise convection within our system, we must first determine the spatial distribution of velocities within the experimental volume. As with the particle density field determination detailed above, we begin by subdividing the system into a series of cells. For a given cell within the system, the mean local velocity may be determined simply by summing the velocities corresponding to all particle locations falling within the cell and dividing by the total number of data points located within the

cell. By performing this process for the x-, y- and z-components of velocity individuallty, velocity vector fields such as those shown in the main article may be produced.

In order to produce the velocity fields presented in the main manuscript, our experimental system is divided into a series of three-dimensional cells of dimension $\Delta x \times \Delta y \times \Delta z$. The precise size of the cells is varied between data sets chosen so as to ensure adequate statistics for the number of, and distribution of, data points acquired which, due to various factors such as run length, system material and tracer activity, may vary between experiments, and cannot be known *a priori* when performing experiments. For each cell, the velocities in the x-z plane of interest are calculated, and then averaged over all cells in the y-direction, ensuring data that is representative of the whole system. The x and z velocities v_x and v_z are then normalised by a factor $\sqrt{v_x^2 + v_z^2}$ such that they may be plotted as unit vectors showing the direction of convection within the system. The plotting as unit vectors ensures that the direction of convection is easily visible – when plotted as unnormalised vectors, the varying lengths of the arrows representing the varying particle velocity can make the direction of convection hard to distinguis in low-velocity regions. Note that the convection velocities (see section 1.4, below) are, however, calculated using unnormalised velocities.

1.3 S1.3: Granular Temperature Fields

The granular temperature, also known as the 'fluctuant kinetic energy' of a system may be defined as:

$$T_g = \frac{1}{2} m \langle c^2 \rangle \tag{3}$$

where $c = |v - \bar{v}|$. Using PEPT data, the granular temperature within the ith cell of our system may be determined as:

$$T_g^i = \frac{1}{2} m \frac{\sum_{j=0}^{j=N_i} (v_i^j - \bar{v}_i)^2}{N_i} \tag{4}$$

where $\bar{v}_i$ is the mean velocity of cell i determined as described in section 1.2, and N_i is the total number of particle locations falling within the cell. By repeating this process for all cells within a system, two-dimensional granular temperature profiles such as those shown in the main text may be produced.

1.4 S1.4: Convection Velocities

In order to determine a value for the mean convective velocity of our systems, we begin by obtaining (unnormalised) 2D, depth-averaged velocity fields as described in section 1.2. We then determine the vertical centre of convection of the system, which corresponds to the height at which the minimum in $|v_x|$ occurs (see Fig. 1).

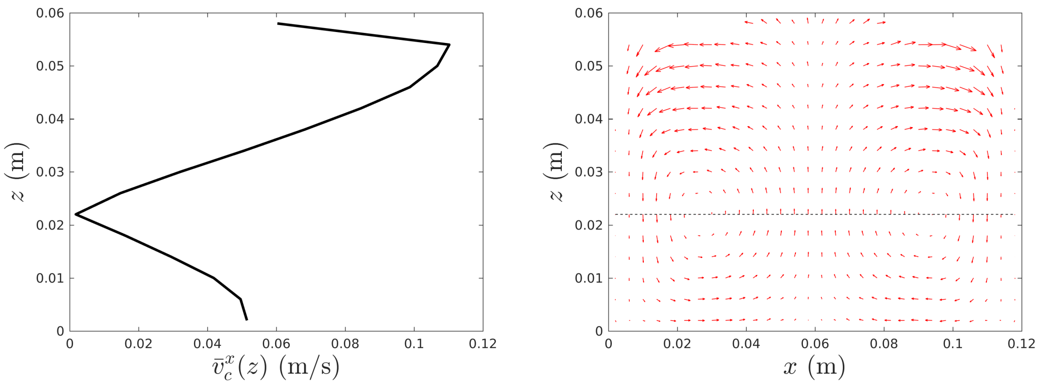

Figure 1. *Left:* Variation with height, z, of the mean horizontal component of the convective velocity, $\bar{v}_c^x(z)$. *Right:* Full convectiove velocity field with the minimum-velocity height superimposed (dotted line).

Since, at the vertical centre of convection, we may assume all convective velocity to be in the vertical direction, and we know that the mass flow rate within the system must be conserved, we may compute the mean convective flow rate, $\bar{v}_c$, of the system, following the methodology of Hsiau and Chen[4], simply as the mean magnitude of the z-component velocity averaged over all cells lying at this height.

1.5 S1.5:Additional quantities

While the above sections detail the manners in which the quantities relevant to the present work may be determined, a number of other valuable parameters may additionally be extracted from PEPT data. For further information regarding the manners in which these other quantities may be computed, and for more information regarding the PEPT technique in general, please refer to our references[1,2,5–8].

1.6 S1.6: Reliability and Repeatability of Experiments

In order to ensure the reliability, generality and repeatability of our experimental results, a two-step process is undertaken. Firstly, all experimental data sets are repeated. Repeat experiments are conducted on different days (to ensure an independence from precise humidity values and other imperfectly controllable external variables which may vary from day to day[*]) and in inverse order (to ensure that wear on the system and/or particles does not influence our results).

Secondly, in order to ensure that our experimental runs are long enough to produce suitable statistics, each data set of duration t_{full} is divided into two non-overlapping partial data sets each of length $t_{partial} = \frac{1}{2}t_{full}$. Three one-dimensional packing density profiles (the simplest spatially-variant whole-field parameter extractable from PEPT data) are then calculated from the three data sets – one each for the full data set and each partial set – using the same cell size (see section 1.1). The full-data and partial-data profiles are then compared against one another point-by-point, allowing a mean percentile error to be calculated. Note that only points falling within the bulk of the medium (here defined as twice the vertical centre of mass of the system – a measure shown viable in previous studies[7]) are considered. If the mean percentile error calculated in this manner lies within 5% for both partial data sets, the data is assumed consistent, i.e. the experiment has been run for a suitably long time.

Once adequate statistics within each repeated experiment have been established, a similar cross-comparison of said repeated experiments is then conducted to ensure reproducibility.

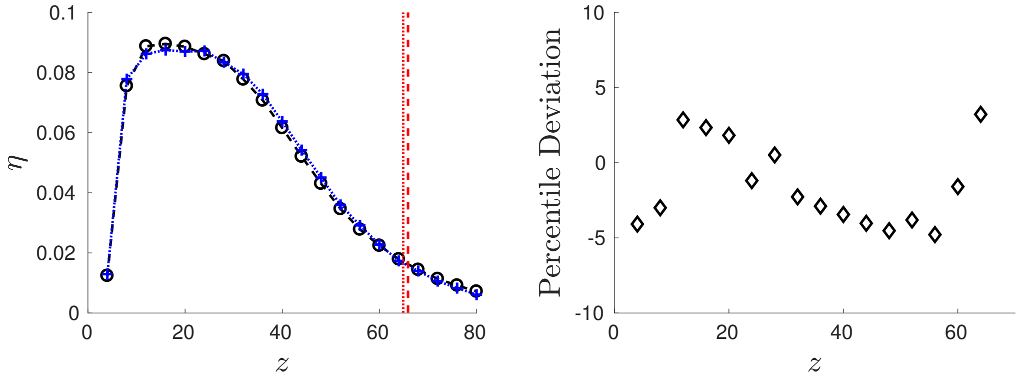

Figure 2. *Left:* Comparison of 1D density profiles produced from two identical repeat experiments performed in a flat, PMMA-walled container with 3mm diameter Nylon particles. The vertical lines shown represent the height of the 'bulk' of the system, calculated as described in the main text of this section. *Right:* The variation with height of the peercentile deviation between the two repeat experiments. The example shown carries a mean percentile error of 2.8%.

2 S2: Discrete Element Method Simulations

In this work, we employ discrete element method (also known as 'discrete *particle* method', DPM) simulations to computationally model our system.

While the system modelled is described in the main text, we discuss here in greater detail the specific algorithms used to model the behaviour of the simulated particles and housing geometry, including the relevant normal and frictional force laws employed.

2.1 S2.1: Simulation Details

The simulation data utilised in this paper is acquired using the *MercuryDPM* discrete element method software package[9–12]. In the current study, interactions between particles are modelled through the implementation of a frictional spring-dashpot model

[*]Note that, due to the large particle size used in our experiments, and the temperature-controlled nature of the laboratory used to perform experiments, such matters are highly unlikely to affect our results in any significant manner.

with linear elastic and dissipative contributions, the relevant normal and tangential forces being calculated, respectively, as[13,14]:

$$f_{ij}^{n} = k^{n}\delta_{ij}^{n}\hat{\mathbf{n}}_{ij} - \zeta^{n}\mathbf{v}_{ij}^{n} \tag{5}$$

and

$$f_{ij}^{t} = -k^{t}\delta_{ij}^{t} - \zeta^{t}\mathbf{v}_{ij}^{t} \tag{6}$$

Here, the symbols $\mathbf{v}_{ij}^{n}$ and $\mathbf{v}_{ij}^{t}$ represent, respectively, the normal and tangential components of (relative) velocity between a given pair of interacting particles; δ_{ij}^{t} is the elastic tangential displacement, whose definition and significance are described in detail in reference[15]); k^{n} and ζ^{n} represent, respectively, the relevant spring constant and damping constants, whose values are determined as:

$$k^{n} = m_{ij}\left[\left(\frac{\pi}{t_c}\right)^{2} - \left(\frac{\log\varepsilon}{t_c}\right)^{2}\right] \tag{7}$$

$$\zeta^{n} = -2m_{ij}\left(\frac{\log\varepsilon}{t_c}\right) \tag{8}$$

In the above, ε is a (user-defined) restitution coefficient, and t_c is the contact time[16], whose value is also determined by the user. In the present work, t_c is chosen so as to give a particle stiffness of 1.5×10^{5} N/m, a value found to provide a realistic degree of overlap (δ_{ij}^{n}) between colliding particles.

In the above equations, the variable m_{ij} corresponds to the reduced mass of a pair of colliding particles i and j, and can be calculated as:

$$m_{ij} = \frac{m_i m_j}{m_i + m_j} \tag{9}$$

The *tangential* spring and damping constants are calculated from their normal counterparts as $k^{t} = \frac{2}{7}k^{n}$ and $\zeta^{t} = \zeta^{n}$.

We implement the Coulomb friction law by applying a static yield criterion which acts to truncate the magnitude of δ_{ij}^{t} at a value equal to μf_{ij}^{n}, such that the inequality $f_{ij}^{t} \leq \mu f_{ij}^{n}$ is fulfilled

Finally, we consider the force of gravity, which acts uniformly on all particles within the system in a direction along the negative z-axis as defined in the main text.

The force relations obtained above are integrated in time using a Velocity-Verlet time-stepping algorithm[17] with a step size $\delta t = \frac{tc}{50}$ to model the evolution of the velocities and positions of the particles within the system.

2.2 S2.1: Validation of Simulation Model

For all experimental cases studied, simulations were performed implementing identical system parameters, in order to assure a qualitative match in the behaviours of interest between simulation and experiment – i.e. that our numerical systems are representative of our ‘real’, physical experiments. Specifically, it was ensured that the number and orientation and positioning of the convection rolls, where present (and the absence of convection where relevant) showed a direct correspondence for an identical parameter set. In Fig. 3, we show equivalent numerical and experimental data sets for the 4 main experimental cases discussed in the main text: flat, dissipative walls (normal convection), flat walls with $\varepsilon_{wall} = \varepsilon_{particle}$ (zero convection), oscillating sawteeth with upward orientation (inverse convection) and oscillating sawteeth with downward orientation (inverse convection). As can be seen from the images provided, our simulations indeed behave as expected based on our experimental results.

References

1. Parker, D., Forster, R., Fowles, P. & Takhar, P. Positron emission particle tracking using the new birmingham positron camera. *Nucl. Instruments Methods Phys. Res. Sect. A: Accel. Spectrometers, Detect. Assoc. Equip.* **477**, 540–545 (2002).
2. Wildman, R., Huntley, J., Hansen, J.-P., Parker, D. & Allen, D. Single-particle motion in three-dimensional vibrofluidized granular beds. *Phys. Rev. E* **62**, 3826 (2000).

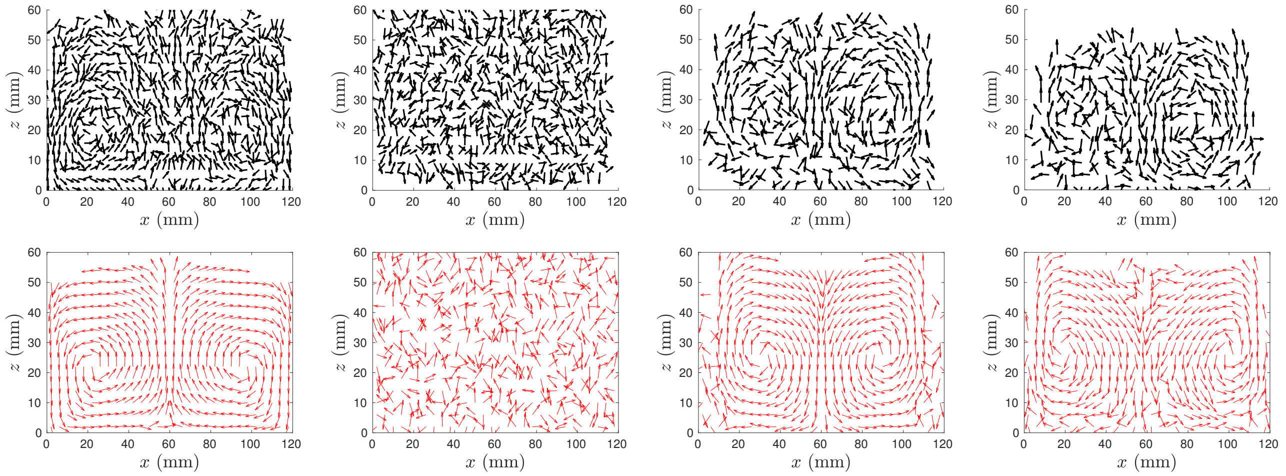

Figure 3. Comparison of experimental (above, black arrows) and numerical (below, red arrows) results for equivalent systems, namely – from left to right – bounded by dissipative PMMA sidewalls, relatively elastic steel sidewalls, upward-oriented oscillating sawteeth and downward-oriented oscillating sawteeth.

3. Wildman, R., Huntley, J. & Parker, D. Convection in highly fluidized three-dimensional granular beds. *Phys. review letters* **86**, 3304 (2001).

4. Hsiau, S. & Chen, C. Granular convection cells in a vertical shaker. *Powder Technol.* **111**, 210–217 (2000).

5. Parker, D., Broadbent, C., Fowles, P., Hawkesworth, M. & McNeil, P. Positron emission particle tracking-a technique for studying flow within engineering equipment. *Nucl. Instruments Methods Phys. Res. Sect. A: Accel. Spectrometers, Detect. Assoc. Equip.* **326**, 592–607 (1993).

6. Windows-Yule, C., Weinhart, T., Parker, D. & Thornton, A. Influence of thermal convection on density segregation in a vibrated binary granular system. *Phys. Rev. E* **89**, 022202 (2014).

7. Windows-Yule, C., Weinhart, T., Parker, D. & Thornton, A. Effects of packing density on the segregative behaviors of granular systems. *Phys. Rev. Lett.* **112**, 098001 (2014).

8. Wildman, R. & Parker, D. Coexistence of two granular temperatures in binary vibrofluidized beds. *Phys. Rev. Lett.* **88**, 064301 (2002).

9. MercuryDPM.org.

10. Thornton, A. R., Weinhart, T., Luding, S. & Bokhove, O. Modeling of particle size segregation: Calibration using the discrete particle method. *Int. J. Mod. Phys. C* **23** (2012).

11. Thornton, A. R. *et al. DEM 6: Proc. 6th Int. Conf. on Discret. Elem. Methods Relat. Tech.* 393 (2013).

12. Thornton, A. R., Weinhart, T., Ogarko, V. & Luding, S. Multi-scale modeling of multi-component granular materials. journal computer methods in materials science. *Comput. Methods Mater. Sci.* **13**, 1–16 (2013).

13. Walton, O. R. Application of molecular dynamics to macroscopic particles. *Int. J. Eng. Sci.* **22**, 1097–1107 (1984).

14. Luding, S. Introduction to discrete element methods: basic of contact force models and how to perform the micro-macro transition to continuum theory. *Eur. J. Environ. Civ. Eng.* **12**, 785–826 (2008).

15. Weinhart, T., Thornton, A. R., Luding, S. & Bokhove, O. Closure relations for shallow granular flows from particle simulations. *Granul. matter* **14**, 531–552 (2012).

16. Luding, S. & McNamara, S. How to handle the inelastic collapse of a dissipative hard-sphere gas with the tc model. *Granul. Matter* **1**, 113–128 (1998).

17. Allen, M. P. Introduction to molecular dynamics simulation. *Comput. Soft Matter: From Synth. Polym. to Proteins* **23**, 1–28 (2004).
